# Human CD8 T cells are susceptible to TNF-mediated activation-induced cell death

**DOI:** 10.7150/thno.41646

**Published:** 2020-03-15

**Authors:** Itziar Otano, Maite Alvarez, Luna Minute, María Carmen Ochoa, Itziar Migueliz, Carmen Molina, Arantza Azpilikueta, Carlos E. de Andrea, Iñaki Etxeberria, Miguel F. Sanmamed, Álvaro Teijeira, Pedro Berraondo, Ignacio Melero

**Affiliations:** 1 Program of Immunology and Immunotherapy, Cima Universidad de Navarra, Pamplona, Spain.; 2 Navarra Institute for Health Research (IDISNA), Pamplona, Spain.; 3 Centro de Investigación Biomédica en Red de Cáncer (CIBERONC), Madrid, Spain.; 4 Department of Pathology, Clínica Universidad de Navarra, Pamplona, Spain.; 5 Department of Histology and Pathology, Universidad de Navarra, Pamplona, Spain.; 6 Department of Oncology, Clínica Universidad de Navarra, Pamplona, Spain.; 7 Department of Immunology and Immunotherapy, Clínica Universidad de Navarra, Pamplona, Spain.

**Keywords:** TNF, AICD, mitochondria hyperpolarization, DNA damage

## Abstract

Activation-induced cell death (AICD) is a complex immunoregulatory mechanism that causes the demise of a fraction of T-lymphocytes upon antigen-driven activation. In the present study we investigated the direct role of TNF in AICD of CD8 T lymphocytes.

**Methods**: Human peripheral mononuclear cells were isolated from healthy donors and fresh tumor-infiltrating lymphocytes were obtained from cancer patients undergoing surgery. T cells were activated with anti-CD3/CD28 mAbs or with a pool of virus peptides, in combination with clinical-grade TNF blocking agents.

**Results**: A portion of CD8 T cells undergoes apoptosis upon CD3/CD28 activation in a manner that is partially prevented by the clinically used anti-TNF agents infliximab and etanercept. TNF-mediated AICD was also observed upon activation of virus-specific CD8 T cells and tumor-infiltrating CD8 T lymphocytes. The mechanism of TNF-driven T cell death involves TNFR2 and production of mitochondrial oxygen free radicals which damage DNA.

**Conclusion**: The use of TNF blocking agents reduces oxidative stress, hyperpolarization of mitochondria, and the generation of DNA damage in CD8 T celss undergoing activation. The fact that TNF mediates AICD in human tumor-reactive CD8 T cells suggests that the use of TNF-blocking agents can be exploited in immunotherapy strategies.

## Introduction

Activation-induced cell death (AICD) is a regulated apoptotic process probably involved in the control of the intensity of T cell-mediated immune responses thereby avoiding excessive clonal expansions 1-3. It is not only an essential mechanism to promote central immune tolerance inducing apoptosis for negative selection during thymocyte development 4, but it has also been proposed to play an important role in peripheral tolerance to self-antigens 5,6. In mature T cells, a strong and chronic activation via T cell receptors may result in T cell hyperactivation leading to AICD 7,8. This form of programmed cell death is mediated by surface receptors with an intracellular death domain and is executed by caspases. Among AICD-inducing receptors, FasL/CD95 is known to play an important role in T cell homeostasis and in the maintenance of immunological tolerance 9-11. FasL is upregulated on activated T cells and can mediate activation-induced cell death (AICD), which physiologically eliminates activated T cells presumed to be no longer needed, with important implications for health and disease including autoimmunity and cancer 6,12-14.

Following activation, multiple T lymphocyte subsets produce TNF and lymphotoxin β. T cells express two types of lymphocyte receptors mainly TNFR2 and weakly TNFR1 15. TNFR1 encompasses a cytoplasmic death domain that recruits the adaptor proteins TNFR1-associated domain protein (TRADD) and Fas-associated death domain (FADD) thereby triggering the activation of the caspase cascade 16. In contrast, TNFR2 recruits TNFR-associated factor 1 (TRAF1) and TRAF2, leading to NF-κB activation controlling the transcription of multiple genes 17. Conceptually, TNF mostly relies on TNFR1 for apoptosis and on TNFR2 for any other function related to T cell function and survival. However, late in the T cell activation process, TNF induces a TNFR2-dependent pathway of cell death in CD8 T cells undergoing activation 18-20. Studies both in TNFR1^-/-^ and TNFR2^-/-^ mice have shown roles for both TNFR1 and TNFR2 in AICD of murine T lymphocytes 18,21. The mechanism of TNFR2-mediated cell death, however, remains elusive. Reportedly, TNF binding to TNFR2 results in apoptosis via RIP-mediated recruitment of FADD 22 or via depletion of TRAF2 and/or cIAPs 23. Although TNFR1 is expressed by almost every mammalian cell type, TNFR2 expression is essentially restricted to immune cells and endothelial cells 15. The consequence of these complex signaling routes in T lymphocytes is that TNF plays a dual role by acting as both an attenuator and mediator of immune responses.

In line with this, two recent reports have shown that blocking TNF in tumor-bearing mice enhances to some extent the antitumor effect of immunotherapy with anti-PD1 mAb or an anti-PD1 + anti-CTLA-4 combinations of mAbs, while at the same time TNF-blockade prevents associated immunopathology 24,25. The effects promoting antitumor immunity could be traced to inhibition of AICD in tumor-reactive CD8 T lymphocytes 24.

We previously published that the apoptotic CD8 T cell death following PBMCs stimulation with anti-CD3/CD28 mAbs was partially inhibited with two neutralizing TNF agents, the TNF-trap etanercept and the monoclonal antibody infliximab 24. In the current study, we sought to elucidate the mechanisms behind this phenomenon in human CD8 T cells and whether they are applicable to human T cells involved in antiviral and antitumor immunity.

## Results

**Figure 1:**
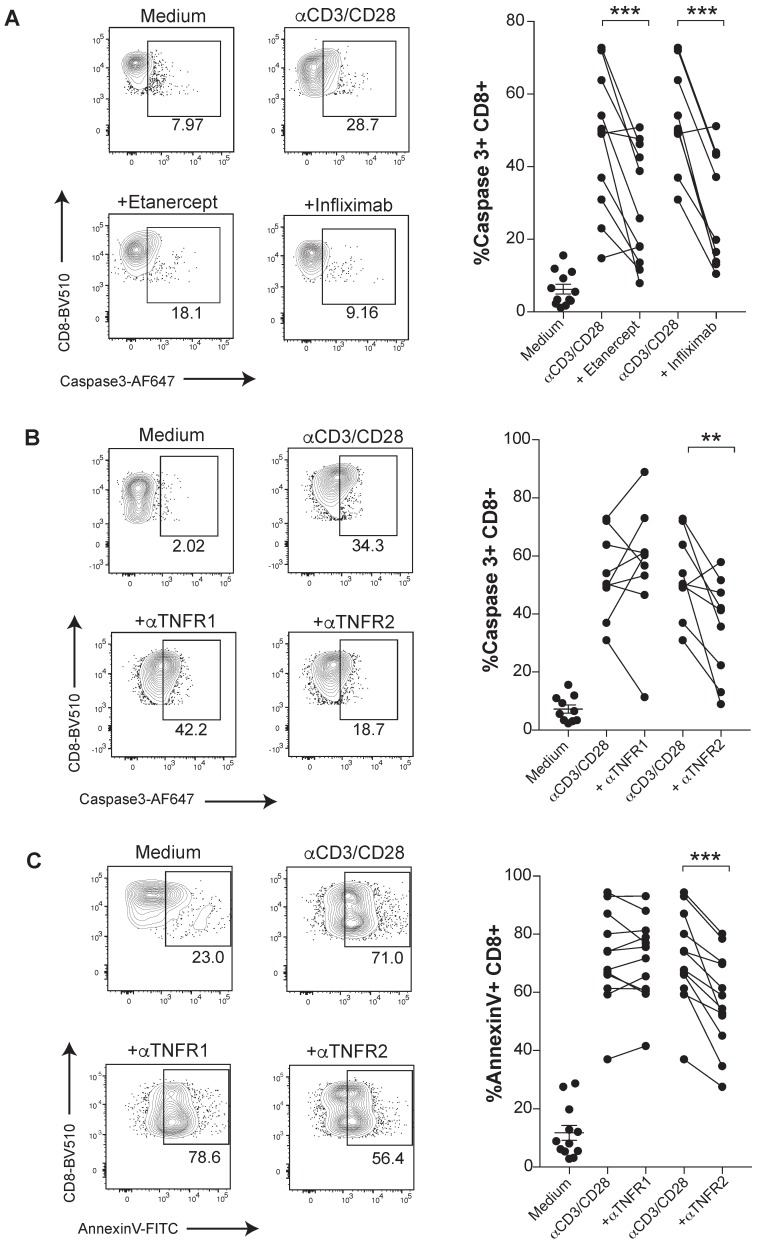
** TNF induces apoptosis through TNFR2 in primary human CD8 T cells undergoing activation.** PBMCs from healthy donors were activated with plate-bound anti-CD3 and soluble anti-CD28 mAbs for seven days in the presence or absence of blocking TNF agents. Apoptosis was measured by active caspase 3 staining of FACs-gated CD8 T cells, in the presence or absence of etanercept (n= 12) or infliximab (n= 10) **(A)**, or selective blocking antibodies against TNFR1 or TNFR2 (n=12) **(B)**. **C**, Surface Annexin V binding was evaluated on FACs-gated CD8 T cells in the indicated culture conditions testing TNFR1 or TNFR2 blockade (n=12). CD8 T cells were pre-gated on Live+/CD3+/CD4-. P values were calculated using a paired two-tailed t-test.

The susceptibility of activated human CD8 T cells to TNF-induced cell death triggered by anti-CD3/CD28 mAbs stimulation was confirmed in terms of a stronger proteolytic activation of caspase 3 (Figure 1A). Caspase 3 activation was mitigated under TNF blockade with etanercept and infliximab (Figure 1A). To determine which receptor was involved in the TNF-mediated AICD, we used specific blocking antibodies against either TNFR1 or TNFR2. We found a significant reduction of activated caspase 3 and surface Annexin V levels when CD8 T cells were treated with anti-TNFR2 mAb but not upon incubation with anti-TNFR1 mAb. Thus, in this cell-culture experimental system, TNF-mediated AICD is mainly dependent on TNFR2 (Figure 1B-C). In contrast, an inhibition of apoptotic activity by the anti-TNFR1 mAb on TNF-susceptible L929 cell line was documented (data not shown).

**Figure 2:**
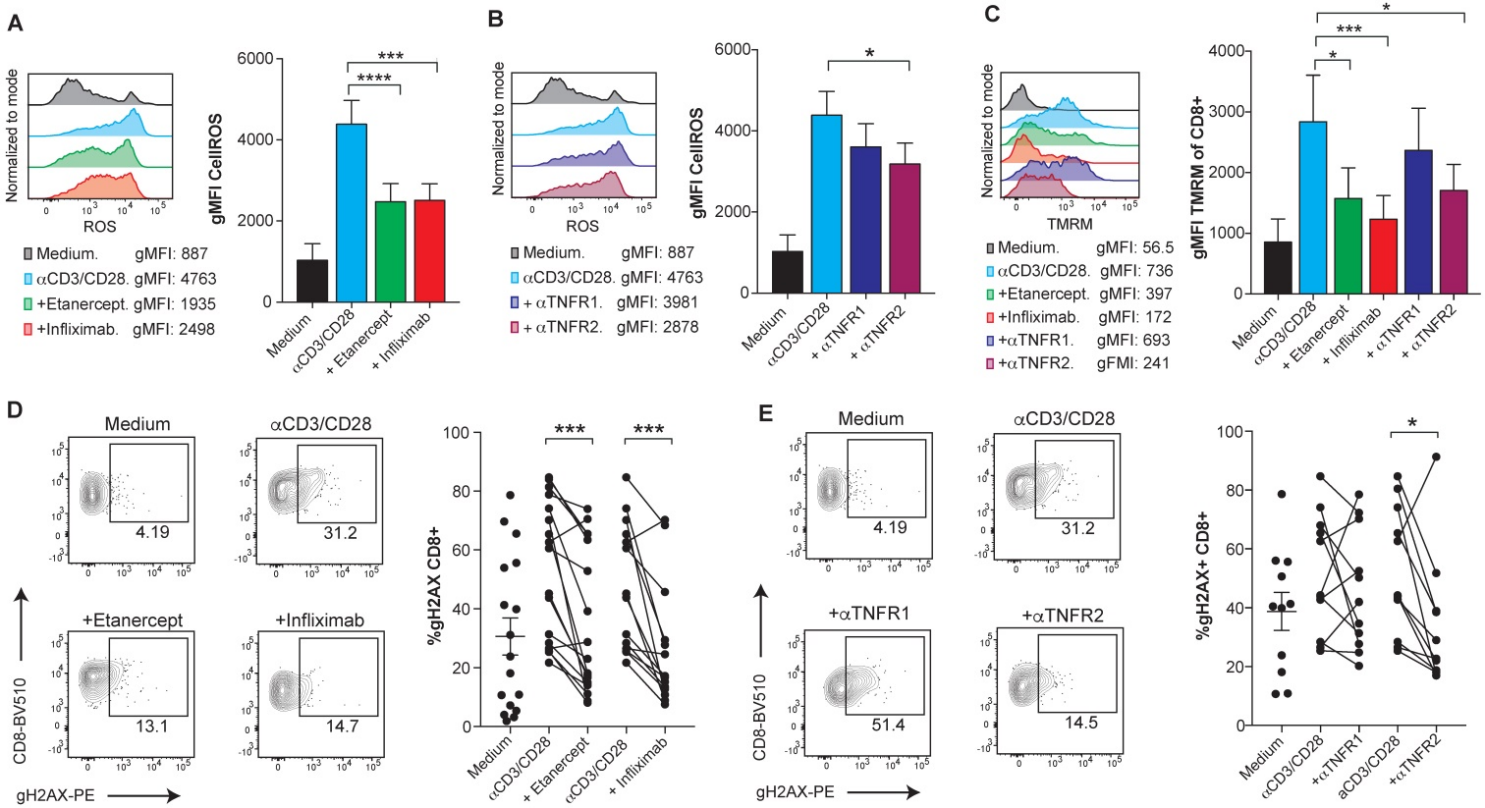
** TNFR2 stimulation on human CD8 T cells increases ROS production via mitochondrial hyperpolarization and causes DNA damage.** PBMCs from healthy donors were activated with plate-bound anti-CD3 and soluble anti-CD28 mAbs for seven days in the presence or absence of blocking TNF agents. Flow cytometry assessment of intracellular ROS geometric mean fluorescence in FACs-gated CD8 T cells stimulated in the presence or absence of etanercept or infliximab **(A)**, or selective blocking antibodies against TNFR1 or TNFR2** (B)** (n=8). **C**, Flow cytometry measurement of TMRM geometric mean fluorescence of FACs-gated CD8 T cells that were stimulated in the presence or absence of TNF blocking agents (n=8). DNA damage was measured by intracellular staining of phosphorylated gH2AX on FACs gated CD8 T cells in the presence or absence of etanercept (n=17) or infliximab (n=14) **(D)**, or selective blocking antibodies against TNFR1 or TNFR2 **(E)** (n=12). CD8 T cells were pre-gated on Live+/CD3+/CD4-. Data for A-C are mean ± s.e.m. and P values were calculated using one-way ANOVA with Tukey's multiple comparsisions test. In D and E, the samples were compared by a paired two-tailed t-test.

Reportedly, the mechanism that leads to TNF-induced cell death is at least in part mediated by the accumulation of intracellular reactive oxygen species (ROS), mainly produced by the mitochondria 26,27. Using flow cytometry analysis, we observed elevated levels of intracellular ROS upon anti-CD3/CD28 activation of human CD8 T cells that were reduced upon TNF blockade and clearly decreased by anti-TNFR2 blocking mAb (Figure 2A-B). Moreover, blockade of TNF reduced the levels of mitochondrial transmembrane potential (ΔΨm) in CD8 T cells, as measured by TMRM, a lipophilic cationic dye that accumulates in the mitochondrial matrix in proportion to the magnitude of ΔΨm electronegativity 28 (Figure 2C). Enhancement of mitochondrial features, including transmembrane potential, is also observed in the case of another TNFR family member, 41BB (TNFR9) 29. Attenuation of mitochondrial polarization by TNF blockade explains reduced ROS production as a byproduct of the electron transport chain. The assessment of TNF-induced DNA damage was measured by the detection of phosphorylated Histone H2AX (gH2AX). Flow-cytometry-assessed levels of gH2AX in CD8 T cells showed that TNF blockade decreases DNA damage in activated human CD8 T cells in a TNFR2 dependent manner (Figure 2D-E). Accordingly, oxidative stress and the DNA damage response, mechanistically link T-cell activation to apoptotic death.

In contrast, T cell effector functions were not affected upon TNFR1 or TNFR2 blockade, shown as the expression levels of CD25, PD-1, Ki67, Granzyme B and IFN-γ production of CD8 T cells after stimulation with anti-CD3/CD28 mAbs (Figure S1 A-E). Moreover, redirected cytolytic activity of CD8 T cells against a tumor cell line in the presence of anti-CD3-Epcam BiTE was not compromised by either TNFR1 or TNFR2 blockade (Figure S1F).

**Figure 3:**
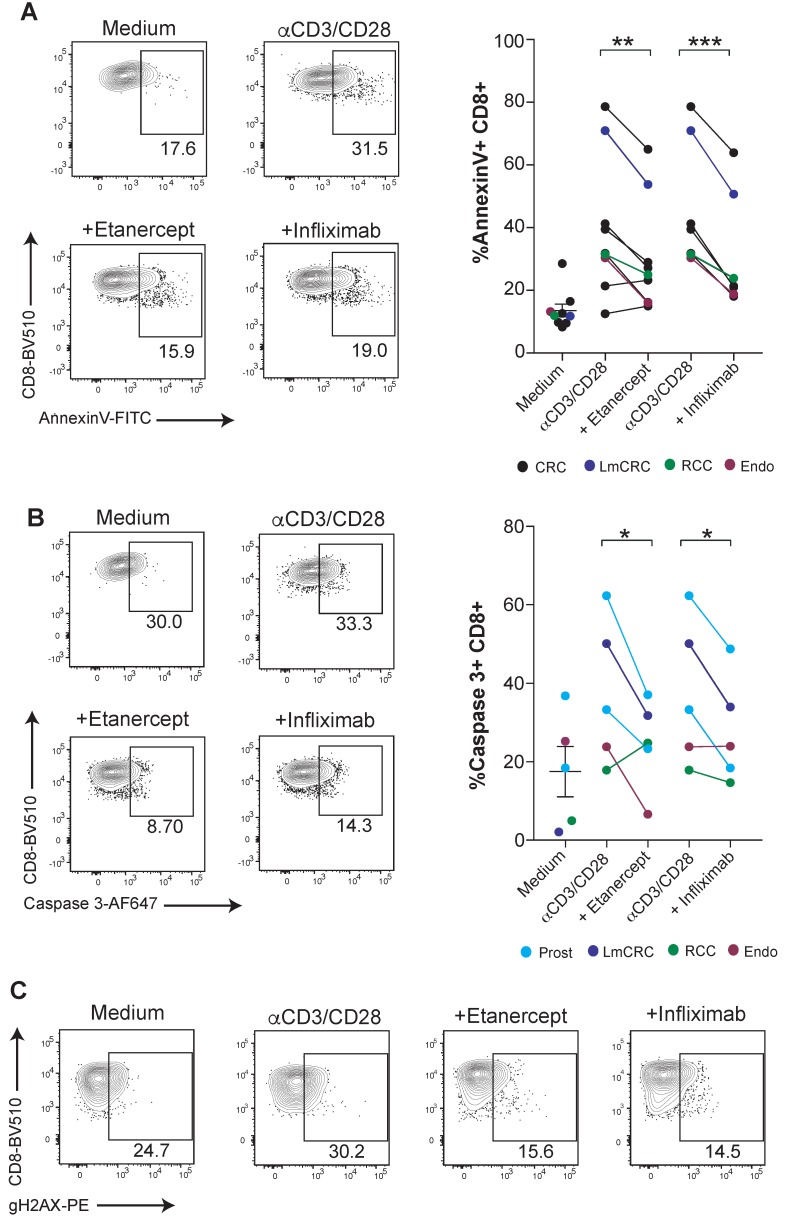
** TNF blockade attenuates AICD in human CD8 tumor-infiltrating lymphocytes.** Fresh cell suspensions from surgical tumor specimens were activated with plate-bound anti-CD3 and soluble anti-CD28 mAbs for 4 days in the presence or absence of anti-TNF blocking agents. The type of tumor is color-coded and indicated in the figure legend (CRC: colorectal carcinoma, LmCRC: colorectal carcinoma liver metastasis, RCC: renal cell carcinoma, Endo: endometrial adenocarcinoma, Prost: prostate carcinoma). Apoptotic cell death was measured by surface Annexin V binding **(A)** and active caspase 3 intracellular staining **(B)** on tumor-infiltrating CD8 T cells. Data for A; anti-CD3/CD28 + etanercept (n = 9); anti-CD3/CD28 + infliximab (n = 7). Data for B; (n = 5). **C**, representative dot plots of phosphorylated gH2AX on FACs-gated CD8 T cells from an endometrial cancer in the cultures set up. CD8 T cells were pre-gated on Live+/CD45-/CD3+/CD4- . P values were calculated using a paired two-tailed t-test.

CD8 T-cell mediated immunity is especially relevant for the defense against malignancies and viral infections in humans. Next, we tested if human tumor-infiltrating lymphocytes (TILs) were susceptible to TNF-mediated cell death. For this purpose, fresh TILs obtained from a number of surgical specimens of a variety of cancers were stimulated with anti-CD3/CD28 mAbs in combination with TNF blocking agents. We found that etanercept and infliximab downregulated the surface binding of Annexin V on CD8 T cells and caspase 3 activation (Figure 3A-B). Moreover, tumor-infiltrating CD8 T cells displayed diminished TNF-induced DNA damage, as measured by gH2AX (Figure 3C). These results highlight the potential relevance of TNF-mediated AICD for hindering immunotherapy in cancer patients.

**Figure 4:**
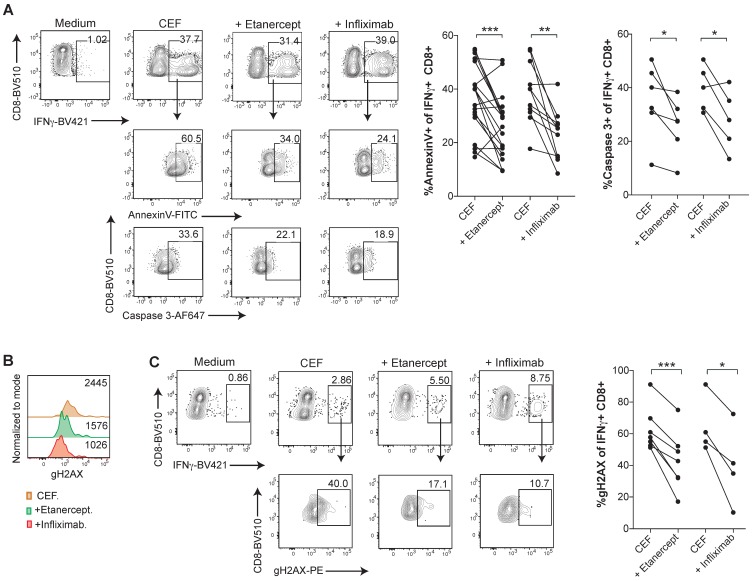
** Human CD8 T cells specific for CMV, EBV or Influenza virus are susceptible to TNF-mediated AICD**. PBMCs from healthy donors were stimulated for 7 days with a pool of MHC-I restricted peptides from CMV, EBV and Flu virus antigens, in the presence or absence of anti-TNF-blocking agents. **A**, analysis of surface Annexin V binding and activated caspase 3 staining of intracellular IFN-γ+ CD8+ T cells upon peptide re-stimulation in the presence or abscence of infliximab or etanercept. Data for Annexin V binding: CEF + etanercept (n=17); CEF + infliximab (n=12). Data for activated caspase 3 staining; CEF + etanercept (n=6); CEF + infliximab (n=5). **(B and C)**, flow cytometry assesment of intracellular staining of phosphorylated gH2AX **(B)** and percentage **(C)** of IFN-γ+ CD8+ T cells in the cultures set up in the presence or absence of entanercept (n=7) or infliximab (n=4). CD8 T cells were pre-gated on Live+/CD3+/CD4-. P values were calculated using a paired two-tailed t-test.

In order to investigate TNF-mediated cell death in an antigen-specific manner in virus-specific CD8 T cell responses, PBMCs were expanded with a pool of viral peptides containing HLA class I-restricted peptides derived from Cytomegalovirus, Epstein-Barr virus, and Influenza virus (CEF) antigens. CEF-specific T cells, identified and gated as IFN-γ^+^ CD8^+^ T cells upon incubation with the peptide pools, showed high levels of Annexin V binding and active caspase 3, whereas TNF blockade with etanercept or infliximab significantly reduced lymphocyte death (Figure 4A-B). Similarly, CEF-specific CD8 T cells displayed reduced levels of gH2AX staining in the presence of TNF neutralizing agents (Figure 4C-D).

In summary, our results support the notion in humans that some virus-reactive and tumor-infiltrating CD8 T cells undergo AICD in a TNF-dependent fashion. The use of TNF blocking agents reduces oxidative stress, hyperpolarization of mitochondria, and the generation of DNA damage, features that precede the demise of some activated CD8 T cells. Our observation is in line with other studies showing that AICD in CD8 T cells is mediated by TNFR2 18,19, whereas other groups have shown that TNFR1 deficient T cells are protected to some extent from TNF-mediated cell death 21,30. It is likely that the involvement of each TNF receptor in AICD is dependent on the time elapsed since the occurrence of T cell activation and on tissue context. Recent evidence has been published suggesting that TNFR2 stimulation with agonist antibodies is costimulatory for T cells 31,32. However, under intense signal-1 conditions, it could be derived into AICD.

## Discussion

Altogether, these data in cultured human CD8 T cells reveal that the use of TNF blocking agents could improve the viable fate of tumor- and antigen-specific CD8^+^ T cells following cognate antigen encounter. Our group and others have shown 21,24,33 that blocking TNF in combination with immune checkpoints facilitates an increase in CD8 T cell numbers and viability in the tumor microenvironment and tumor draining lymph nodes, enhancing anti-tumor efficacy while paradoxically reducing immune related adverse effects (irAEs). In a recent report, cancer patients treated with immune checkpoint blockade agents having to receive infliximab for irAE management did not show inferior outcomes of their malignancies 34. An ongoing phase I clinical trial (NCT03293784) is evaluating the safety and tolerability of treating metastatic melanoma patients with immune checkpoint blockade in combination with the anti-TNF blocking agents infliximab and certolizumab and is expecting to mitigate or avoid irAEs while augmenting antitumor efficacy. Our study showing inhibition of AICD in human CD8 T cells upon TNF blockade points in this direction.

## Conclusions

Our study conclusively shows in human CD8 T cells the TNF-mediated AICD phenomena previously described in mouse models and highlights that they can be exploited in immunotherapy since they are relevant for virus-specific and tumor-infiltrating T lymphocytes. Importantly, such effects could be interfered with using clinically available agents such as infliximab and etanercept.

## Materials and methods

### Subjects and sample collection

This study was approved by the Regional Ethics Committee and written informed consent was obtained from all participants. A total of 25 healthy volunteers participated in the study. PBMCs were separated using Ficoll-Paque Plus (GE Healthcare) density gradient centrifugation. Fresh and sterile tumor tissue samples were obtained from the Department of Pathology of the University Clinic of Navarra, following written, signed, and dated informed consent according to a protocol approved by the institutional ethics committee protocol (2019-76). The characteristics of the patients used in the study are depicted below:

### Activation-induced cell death assessment in PBMCs

PBMCs from healthy donors were isolated by Ficoll-Hypaque gradient separation (Lymphoprep). PBMC were stimulated with 0.5 µg/ml of plate-bound anti-CD3 mAb (OKT-3; eBioscience) and 1 µg/ml of soluble anti-CD28 mAb in RPMI-10% FBS supplemented with IL-2 (20 U/ml, Proleukin) in the presence or absence of 10 μg/ml of anti-hTNF (Infliximab, Janssen), 4 μg/ml of Etanercept (Sandoz Farma), 1 μg/ml anti-TNFR1 (clone: 55R-170, ThermoFisher Scientific) or 1 μg/ml anti-TNFR2 (clone: 2222.311, ThermoFisher Scientific). Seven days later, cells were re-plated into fresh anti-CD3/CD28 mAbs coated plates and cultured overnight in the same conditions. Cells were analyzed by multicolor flow cytometry (BD FACSCanto™ II system; Zombie NIR, anti-CD3 PeCy7 BD, anti-CD8 BV510, anti-CD4-PE, Annexin V-FITC, anti-CD25-BV421, and anti-PD-1 PerCPCy5.5 (BioLegend). All analyses were performed using Flowjo software (Tree Star).

### Activation-induced cell death assessment in viral antigen-specific T cells

Whole PBMCs from healthy donors were stimulated with 0.5 μg/ml CEF extended pool consisting of 32 8-10-mer peptides, each corresponding to a defined HLA class-I restricted epitope from Cytomegalovirus, Epstein-Barr virus and Influenza virus (JPT Peptide Technologies) in the presence of IL-2 (20U/ml) and in the presence or absence of 10 μg/ml of anti-hTNF (Infliximab, Janssen) or 4 μg/ml of Etanercept (Sandoz Farma). On day 7, PBMCs were restimulated using 0.5 μg/ml of CEF peptides under the same conditions and in the presence of brefeldin A (BFA).

### Tumor explants

Tumor tissue was digested using enzymes (1 ng/ml of collagenase IV and 0.1 ng/ml of DNAse I) and mechanical maceration before being passed through a 70μm cell strainer. Cell suspensions were stimulated with 0.5 µg/ml of plate-bound anti-CD3 mAb (OKT-3; eBioscience) and 1µg/ml of anti-CD28 mAb in RPMI-10%FBS supplemented with IL-2 (20 U/ml) in the presence or absence of 10 μg/ml of anti-hTNF (Infliximab, Janssen) or 4 μg/ml of Etanercept (Sandoz Farma). On day 4, cells were restimulated in the same culture conditions.

### Intracellular and intranuclear staining of human PBMCs

Dead cells were excluded using a live/ dead fixable dye staining kit (BioLegend). Cells were stained for surface markers and then fixed and permeabilized with CytoFix/Perm Buffer (BD). Intracellular molecules were detected using anti-IFN-γ-BV421 (BD) and anti-caspase 3-AF647 (BD) and anti-Granzyme B AF647 (BioLegend). For intranuclear staining, the True nuclear transcription buffer set from Biolegend was used according to the manufacturer protocol. Antibody against anti-Ki67 AF488 (BioLegend) was used. All samples were acquired on a BD Canto II. All analyses were performed using Flowjo software (Tree Star).

### Intranuclear staining of gH2AX in human PBMCs

Dead cells were excluded using a live/dead fixable dye staining kit (BioLegend). Cells were stained for surface markers and then fixed with 4% of paraformaldehyde. Cells were then permeabilized with cold Perm III Buffer (BD). Then, gH2AX-PE (BioLegend) was detected by intranuclear staining. All samples were acquired on a BD Canto II. All analyses were performed using Flowjo software (Tree Star).

### Intracellular ROS and TMRM measurement

For analysis of intracellular ROS, PBMCs were incubated with 5 μM of CellROS Deep Red reagent (ThermoFisher) for 30 min at 37°C. Membrane potential was assessed using the potentiometric dye tetramethylrhodamine methyl ester (TMRM; Sigma Aldrich) at a final concentration of 125 ng/ml for 30 min at 37°C. Cells were then stained for live/dead and surface markers. All samples were acquired on a BD Canto II. All analyses were performed using Flowjo software (Tree Star).

### *In vitro* killing assay

*In vitro* real-time killing assays were performed by measuring electric impedance overtime in an Xcelligence Real-Time Cell Analysis Instrument (ACEA). 5x10^4^ HCT116 cells were seeded onto a 16-well plate (ACEA) and cultured overnight in a Xcelligence instrument for cell adhesion and stabilization. After overnight culture, 2.5x10^5^ human primary CD8 T cells were added with 0.5 μg/ml of anti-CD3-Epcam bispecific T-cell engager (BiTE) (Creative Biolabs), in the presence or absence of 1 μg/ml anti-TNFR1 (clone: 55R-170, ThermoFisher Scientific) or 1 μg/ml anti-TNFR2 (clone: 2222.311, ThermoFisher Scientific). Electric impedance was measured every 5 min for 25 h.

### Statistical analysis

Statistical analyses were performed using two-way ANOVA, Student's t-tests and Tukey's tests, as appropriate and indicated in each figure. Significant differences were marked on figures legends as *<0.05, **<0.01 and ***<0.001.

## Supplementary Material

Supplementary figure.

## Figures and Tables

**Table 1:** The characteristics of patients

Gender	Age at surgery	Disease	Stage
Male	87	Renal cell carcinoma (RCC)	pT3NxMx
Male	61	Colorectal carcinoma liver metastasis (LmCRC)	pT3N2M1
Female	80	Colorectal carcinoma (CRC)	pT2N0
Female	80	Colorectal carcinoma (CRC)	pT3N0Mx
Female	71	Colorectal carcinoma (CRC)	pTispN0
Female	62	Sigmoid adenocarcinoma (CRC)	pT2N0
Male	60	Colorectal carcinoma (CRC)	pT3N0
Female	63	Endometrial adenocarcinoma (Endo)	pT1bN0
Male	67	Prostate cancer (Prost)	pT2cN0
Male	57	Prostate cancer (Prost)	pT2bN0

## Abbreviations

AICD: activation-induced cell death
ANOVA: analysis of variance
DNA: deoxyribonucleic acid
irAEs: immune related adverse effects
CTLA-4: Cytotoxic T-Lymphocyte Antigen 4
FADD: Fas-associated death domain
IFNγ: interferon gamma
PBMCs: peripheral blood mononuclear cells
PD-1: programmed cell death protein 1
TMRM: Tetramethylrhodamine, methyl ester
TILs: tumor infiltrating lymphocytes
TNF: tumor necrosis factor
TNFR1: tumor necrosis factor receptor 1
TNFR2: tumor necrosis factor receptor 2
TRADD: TNFR1-associated domain protein
