# Supplementary material for: Human CD8 T cells are susceptible to TNF-mediated activation-induced cell death
Source: Theranostics. 2020 Mar 15;10(10):4481–9. doi: 10.7150/thno.41646 (PMC7150490; doi:10.7150/thno.41646)
Supplement: Supplementary file 1 — Supplementary figure. [file thnov10p4481s1.pdf]

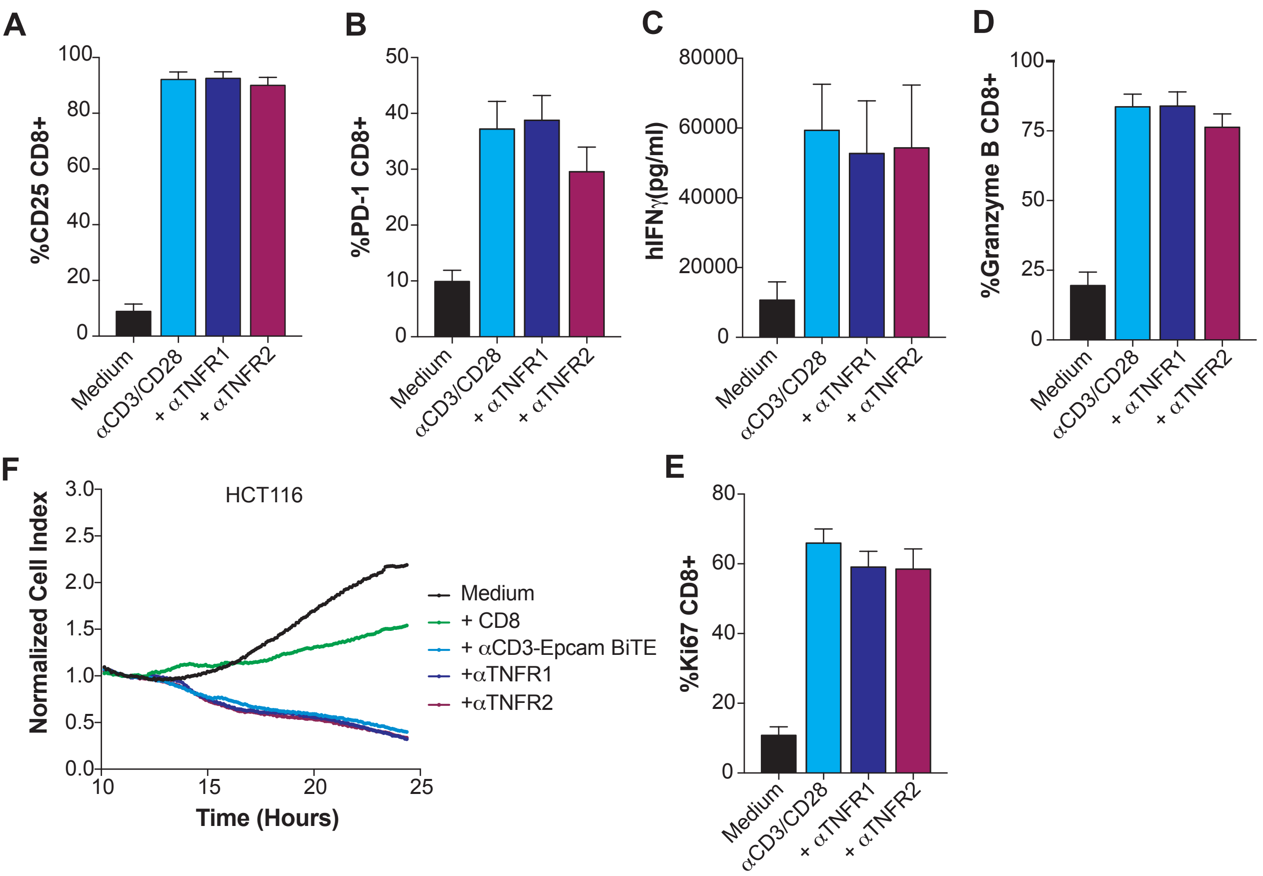

Supplementary Figure 1. **Activation and proliferation of CD8 T cells after TNF blockade with TNFR blocking antibodies.** PBMCs from healthy donors were activated with plate-bound anti-CD3 and soluble anti-CD28 mAbs for seven days in the presence or absence of selective blocking antibodies against TNFR1 or TNFR2. Flow cytometry assessment of surface expression of CD25 (**A**) and PD-1 (**B**) (n=12) on FACs-gated CD8 T cells. **C**, supernatants from the cultures set up in the presence or absence of blocking antibodies against TNFR1 and TNFR2 were analyzed to determine the concentration of soluble IFN-γ levels (n=7). **D**, intracellular levels of Granzyme B (n=8) in FACs-gated CD8 T cells. **E**, real-time lysis of HCT116 tumor cells when cultured with CD8 T cells and anti-CD3-Epcam BiTE, in the presence or absence of selectively blocking antibodies against TNFR1 or TNFR2. Impedance-based tumor-cell cytotoxicity was performed in duplicate wells. **F**, flow cytometry assessment of intracellular levels of Ki67 (n=8) in FACs-gated CD8 T cells.
